# Gel-Trapped Lymphorganogenic Chemokines Trigger Artificial Tertiary Lymphoid Organs and Mount Adaptive Immune Responses *In Vivo*

**DOI:** 10.3389/fimmu.2016.00316

**Published:** 2016-08-22

**Authors:** Yuka Kobayashi, Takeshi Watanabe

**Affiliations:** ^1^The Tazuke-Kofukai Medical Research Institute, Kitano Hospital, Kita-ku, Osaka, Japan

**Keywords:** artificial tertiary lymphoid organs, immune therapeutics, primary immune deficiencies

## Abstract

We previously generated artificial lymph node-like tertiary lymphoid organs (artTLOs) in mice using lymphotoxin α-expressing stromal cells. Here, we show the construction of transplantable and functional artTLOs by applying soluble factors trapped in slow-releasing gels in the absence of lymphoid tissue organizer stromal cells. The resultant artTLOs were easily removable, transplantable, and were capable of attracting memory B and T cells. Importantly, artTLOs induced a powerful antigen-specific secondary immune response, which was particularly pronounced in immune-compromised hosts. Synthesis of functionally stable immune tissues/organs like those described here may be a first step to eventually develop immune system-based therapeutics. Although much needs to be learned from the precise mechanisms of action, they may offer ways in the future to reestablish immune functions to overcome hitherto untreatable diseases, including severe infection, cancer, autoimmune diseases, and various forms of immune deficiencies, including immune-senescence during aging.

## Introduction

It is well established that the function of the immune system may be compromised in a variety of diseases resulting in clinically significant immune deficiencies. Secondary immune deficiencies may be caused by overwhelming microbial infections, surgical removal of secondary lymphoid organs (SLOs), or destruction by tumor invasion leaving the host immune system incapable to mount effective immune responses with often lethal clinical outcomes. The function of the immune system is also severely compromised during aging due to immune senescence. Until today, no effective treatment regimens are available for such conditions. In order to restore the exhausted function of lymphoid tissues, a trial has recently been reported using therapeutic formation of TLOs in tumor-bearing hosts by delivering molecules known to be involved in immune system homeostasis, such as lymphotoxin-α1β2 or LIGHT, which stimulate lymphotoxin-β receptors on tissue-resident stromal cells (1–7). Newly synthesized TLOs appear to be effective to suppress tumor growth under distinct experimental conditions (2, 3, 8). Although TLO formation has been thought to be associated with autoimmune disease progression rather than suppression, TLOs may play beneficial roles by enhancing protective immunity in a variety of disease conditions (8, 9).

Structures of both SLOs and TLOs resemble each other in many ways, including segregated T and B cell compartments, the presence of CD11c^+^ dendritic cells (DCs), networks of fibroblastic reticular cells (FRCs) and follicular DCs (FDCs), and the formation of high endothelial venules (HEV) (10–15). It is well known that TLO neogenesis recapitulates many features of SLO formation involving molecules secreted from lymphoid inducer (LTi) cells, such as lymphotoxin α1β2 and LIGHT (4–7). Rat insulin promoter (RIP)-LTα transgenic mice, which express Lta (lymphotoxin-α) gene driven by the RIP, develop TLOs in pancreatic islets, skin, and kidney (16). Transgenic mice expressing both LTα and LTβ (LTα1β2) under the control of RIP had larger lymphoid tissues with distinct T and B cell areas, well-developed FDC networks, and higher expression of CCL19, CCL21, and CXCL13 chemokines (also referred to as lymphorganogenic chemokines) in the pancreatic islets when compared to transgenic mice expressing only LTα (4).

Overexpression of CCL21 under control of either the thyroglobulin or RIP promoters developed TLOs in the thyroid or in the pancreatic islets, respectively (17, 18). The TLOs also showed PNAd positive HEVs and lymphatic vessels (19–21). Mice expressing CXCL13 under the control of RIP also developed TLOs with distinct B cell follicles and T cell areas in the pancreas (4, 22). Moreover, mice overexpressing CXCL12 in the pancreas showed lymphoid aggregates with accumulation of DCs, B cells, and plasma cells but few T cells (22). Transgenic mice with CCL19 gene expression under control of RIP showed small cell infiltrates composed of lymphocytes and DCs (22). CXCL13 stimulated LTαβ expression in B cells, while CCL19 and CCL21 trigger LTαβ expression in CD4 T cells (22). LTαβ expression was also induced by the stimulation of naive T cells by IL-4 and IL-7 (19).

In tertiary lymphoid organs located in inflammatory tissues in humans, CCL19 and CCL21 are apparently secreted from the smooth muscle actin-positive stromal cells, in close proximity to HEVs (23). Mature DCs and lymph vessels also promote CCL21 expression. The ligand for CXCR4, i.e., CXCL12, contributes to T cell trafficking in lymph nodes and Peyer’s patches together with CCR7 ligands, i.e., CCL19 and CCL21 (24). Another important chemokine for lymphocyte trafficking is CXCL13, which is expressed by stromal cells, including FDCs in germinal centers of SLOs. CXCL13 initiates migration of CXCR5-expressing B cells into follicles and thereby contributes to lymphoid organ formation (25). Importantly, CXCL13 also recruits follicular helper T (T-FH) cell into B cell follicles (26, 27). LTα1β2 is not only expressed by B cells but also by LTi cells thereby further promoting generation of an FDC network, including CXCL13 expression in FDCs (28). These cell/cell interactions driven by lymphorganogenic chemokines stimulate recruitment of B cells and establish a positive feedback loop for B cell follicle homeostasis (29). A crosstalk between lymphotoxin-expressing B cells and FRCs plays a role in promoting B cell follicle formation through activation of B cells expressing type 2 inflammatory cytokines (30). FRC networks containing lymph node-like conduits that promote lymph flow also provide tracks for T cell migration, which is promoted by CCR7 ligands, CCL19 and CCL21. FRCs produce various survival factors for lymphocytes besides CCL19 and CCL21, such as IL-6, IL-7, and B cell survival factor BAFF (31), VEGF (32), and retinoic acid (RA) (33, 34). The conduit system is not only en-sheathed by FRCs and connected to the FRC network but FRCs generate conduits (35). Conduits also link to lymph vessels in draining lymph nodes (see contribution by Nancy Ruddle, this Research Topic). CCL21 secreted from FRCs promotes fluid flow in conduits, which enhances the organization of FRC networks (36). Blocking lymph flow in peripheral lymph nodes downregulates CCL21 and CCL19 gene expression in FRCs. All these data together suggest that the increased lymph flow in inflammatory tissues promotes FRC networks resulting in enhancement of immune cell trafficking, sampling of lymph, and enhancing antigen-specific immune responses. CD4 depletion results in FRC loss because of lack of lymphotoxin-β produced by CD4 T cells. Thus, CD4 T cells appear to play a central role in maintaining lymphoid tissue structure and homeostasis through secretion of lymphotoxin-β (37). Thus, mutual interactions between FRC and CD4 T cells may play a role for formation of TLOs. RA may participate in homing of activated T cells through activation of DCs (38). The interaction of signal regulatory protein α (SIRPα), Ig superfamily protein, expressed on the DCs, and T cells with its counterpart CD47, expressed on non-hematopoietic cells such as lymphoid stromal cells, play crucial roles in T cell homeostasis and formation of T cell area in the spleen white pulp (20), suggesting that interaction between SIRPα and its binding partner CD47 may be required for formation of SLOs as well as TLOs. These data indicate mutual interactions between lymphoid tissue organizer (LTo)-like stromal cells and immune cells, such as T cells, B cells, and DCs and these principles known for SLOs may also apply to TLOs. At inflammatory sites, such as aortic smooth muscle cells adjacent to atherosclerotic plaques (37, 39, 40) and local resident fibroblasts (41) give rise to LTo-like stromal cells, which express chemokines, such as CXCL13, CCL19, and CCL21 upon stimulation with lymphotoxin-αβ through lymphotoxin-β receptor (LTβR).

Appearance of TLOs has been reported during allograft rejection (42–45), indicating that TLOs may play a role in induction of an effective immune response upon alloantigen stimulation and serve as a site for local adaptive immune responses. Transplantation of skin grafts prepared from RIP-LTα transgenic mice into allogeneic splenectomized aly/aly mice, which lack SLOs, resulted in the rejection of skin grafts containing TLOs. Mice, which lack both spleen and peripheral lymph nodes, were resistant against viral infection but they mounted a strong immune defense by generating TLOs (9, 43). These reports indicated that antigen-specific activation of host-derived naive T cells as well as the establishment of memory T cells had taken place in the TLO. Thus, TLOs also play a role as an activation site of naive T cells to effector and memory T cells similarly to SLOs. It has been recently reported that TLO in the artery such as aorta could control aorta immunity and protect against atherosclerosis (39, 40). Taken together, TLOs may organize highly localized immune responses against microbial-derived antigens, tumor-derived antigens, and auto- or alloantigens. It, therefore, appears a promising concept to construct artificial lymph node-like tertiary lymphoid organs (artTLOs) for the treatment of various clinically important diseases (46).

Lymph node FRCs ectopically expressed peripheral tissue antigen (PTA) and directly presented it to naive T cells under steady state as well as inflammatory conditions (47). Moreover, lymph node-resident lymphatic endothelial cells directly present PTA to T cells and mediate peripheral tolerance independently of autoimmune regulator (Aire) (48). These findings suggest that lymph node stromal cells (LTo) appear to be involved in not only the TLO formation but also the maintenance of tolerance to self-antigens in the periphery at adult stage. As discussed above, artery TLO, which emerge in the aorta adventitia adjacent to atherosclerotic plaques, regulate immunity in aorta, and protect against atherosclerosis through LTβR expressed on vascular smooth muscle cells (40). These diverse function displayed by stromal cells suggests that the artTLO may acquire the immunological function when the stromal cells adopt or differentiate into LTo cells. These diverse functions may include not only promotion of antigen-specific protective immune response to treat autoimmune diseases. Therefore, our principal tenet has been to create artTLOs with various and even opposite functions to foster maintenance of immune homeostasis depending on the disease conditions.

We had previously reported synthesis of artTLOs with the ability to induce immune responsiveness *in vivo* by applying lymphoid stromal cells and bone marrow-derived DCs (49–52). The stromal cell line TEL-2 (53), which had been established from neonatal mouse thymus, was transfected with LTα cDNA. The LTα-expressing stromal cells or TEL-2 cells stimulated with LTα-coated beads expressed VCAM-1 and ICAM-1, and secreted lymphorganogenic chemokines, including CCL19, CCL21, and CXCL13. LTα-expressing stromal cells were then mixed with bone marrow-derived DCs (49, 50). The cell suspension was first incorporated into collagen sponges, which were subsequently transplanted into the renal subcapsular space of mice. After 2–3 weeks, lymphocyte-rich cell-aggregates had emerged in the collagen sponges. The resulting structures consisted of clearly segregated clusters of T and B cells, FDCs in B cell follicles, and FRC networks in T cell areas. HEVs, lymph vessels, and germinal center formation upon antigen stimulation were also evident (49). Thus, the grafts were termed as artificial lymph node tissues (aLN) but are more appropriately called *artTLOs*. When the artTLOs were generated in mice that had been preimmunized with antigen, they were capable of inducing a strong secondary immune response *in vivo* upon antigen re-stimulation, as evidenced by the accumulation of effector memory and T-FH cells, as well as antigen-specific memory B cells (50). In addition, the artTLOs were capable of inducing a strong secondary immune response when re-transplanted into naive mice upon immunization with the antigen. Also, re-transplantation of the artTLOs into SCID mice, followed by immunization, let to a robust secondary immune response. The artTLOs as well as spleen cells in SCID mice produced large amounts of antigen-specific high affinity IgG class antibodies consistent with the possibility that somatic hypermutation, germinal center reaction, affinity maturation, and Ig class switching were conducted in the artTLOs (50). Furthermore, the artTLOs appeared to suppress tumor growth when they were transplanted into tumor-bearing mice (51, 52). This was the first proof of principle that artificial lymphoid tissues are transplantable and immunologically active.

Taken together, these previous data led us to develop new strategies to artificially synthesize transplantable and immunologically functional lymphoid tissues/organs in the absence of stromal cells, i.e., LTo cells. We hypothesized that *in vivo* transplantation of a combination of lymphorganogenic chemokines and cytokines as a substitute for LTo cells would be feasible. Here, we report that functionally highly active artTLOs can indeed be generated by applying slow-releasing gels containing lymphotoxin-α1β2 and additional chemokines on a collagen matrix.

## Results

### Preparation of Gels and Formation of artTLOs

**Figure 1:**
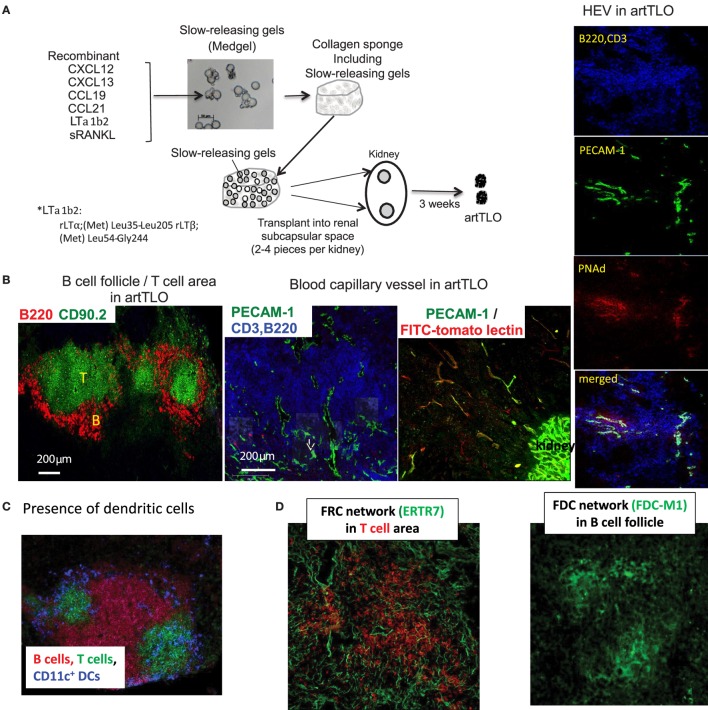
**Strategy to construct functional artTLOs**. **(A)** The slow-releasing gel-beads (Medgel) containing each recombinant chemokine and cytokine protein indicated in the Figure were prepared. Briefly, solution (100 μg/ml) of each soluble recombinant protein (CXCL12, CXCL13, CCL19, CCL21, LTα1β2, and sRANKL) was added to the dry powder of the Medgel in 1.5 ml micro tube, respectively, and was incubated for 2 h at 37°C. Then, Medgels containing each chemokine and LTα1β2 were combined and mixed. The combined gels were incorporated into the collagen sponges as described in the Section “Materials and Methods.” The collagen sponges were then transplanted into the renal subcapsular space of NP-OVA preimmunized Balb/c mouse. After 3 weeks, artTLO had formed. **(B)** Lymphoid cell assembly consisted of segregation of T cell area and B cell follicles. Functional capillaries and lymph vessels were detected. Functional arteries and high endothelial venules (HEV) were well developed. **(C)** Distribution of dendritic cells in T cell area. **(D)** FDC network in B cell follicle and FRC network in T cell area.

Although application of stromal cell lines is an effective strategy for the construction of artificial lymphoid tissues (47, 48), the approach has major limitations in clinical practice. Consequently, we sought to establish a cell-free method. For this purpose, we transplanted collagen sponge scaffolds containing the slow-releasing Medgel beads in which lymphotoxin-α1β2, CCL19, CCL21, CXCL12, CXCL13, and soluble RANK ligand (sRANKL) were trapped (experimental strategy is outlined in Section “Materials and Methods” and legend for Figure 1A). Gel-beads gradually release each protein over extended period of time and are concomitantly resolved by the endogenous collagenase. The collagen sponge containing randomly arranged gel-beads was transplanted into the renal subcapsular space of mice. After 3 weeks, grafts were removed and the resulting cell aggregates were examined by immunofluorescence microscopy. Medgel alone without any chemokine did not give rise to any tissue graft. Although Medgels containing lymphotoxin-α1β2 or any of each recombinant CCL19, CCl21, CXCL12, or CXCL13 chemokine formed more or less of a tertiary lymphoid tissue-like cell mass, referred to as artTLO, as suggested by the previous reports mentioned in the Section “Introduction,” mixtures of gels containing lymphotoxin-α1β2 and four different types of chemokines, CCL19, CCL21, CXCL12, and CXCL13, together with sRANKL gave constantly the most advanced lymphoid structures. They consist of segregated B cell and T cell areas (Figure 1B), DCs in T cell areas (Figure 1C), FDC and FRC networks (Figure 1D), and appearance of HEVs-like structure (Figure 1B, right side). Besides, angiogenesis was prominent in the lymphoid tissues and lymph capillary vessels appeared in the periphery (Figure 1B, middle).

### Memory B and T Cells Are Major Cellular Constituents in artTLO

**Figure 2:**
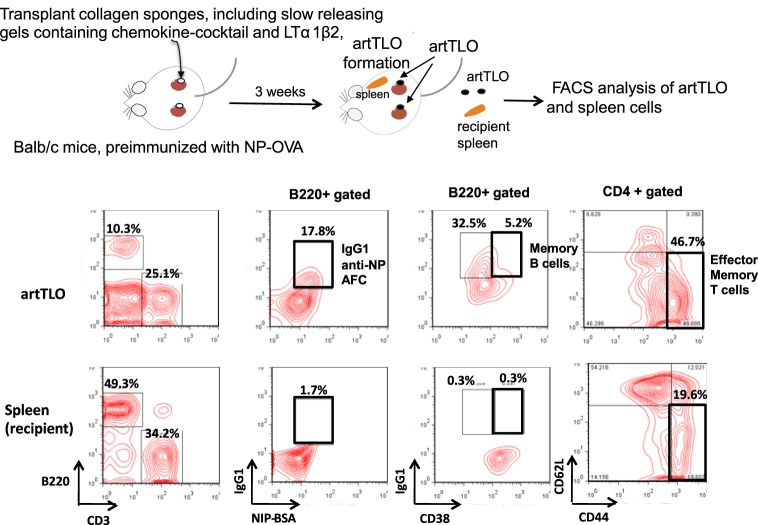
**Accumulation of antigen-specific memory B cells and CD4 positive T cells in artTLOs**. artTLOs were constructed in Balb/c mice, which had been preimmunized with 100 μg NP-hapten conjugated ovalbumin (NP-OVA) in alum as described in the Section “Materials and Methods.” After 3 weeks, artTLO were formed in the subcapsular space of kidney. artTLO and spleen were removed from recipients for flow cytometer analysis. Flow cytometer profiles of artTLO and spleen cells of recipient mice are shown. Note that IgG1 class anti-NP antibody-forming cells as well as memory B cells and effector memory T cells are remarkably enriched in artTLOs, compared to the recipient spleen cells. Representative data are shown from FACS analysis of 10 individual artTLO. Data were highly reproducible.

artTLOs were formed in renal subcapsuler space of Balb/c mice that had been preimmunized with NP hapten-coupled chicken egg albumin (NP-OVA) in alum more than 1 month before artTLO formation was initiated. ArtTLOs and spleens of recipient Balb/c mice were then excised. The artTLOs and spleens were minced and single-cell suspension was prepared from each artTLO or spleen, followed by fluorescence flow cytometer analysis (Figure 2). A large number of anti-NP antibody-secreting cells were detected in artTLO but few in the recipient spleen (the second column from left). Furthermore, B220^+^CD38^+^IgG^+^ memory B cells (third column from left) and CD3^+^CD4^+^CD44^+^CD62L^−^ effector memory T cells (fourth column from left) were highly enriched in artTLOs when compared to recipient spleens. IgG class anti-NP antibody-forming cells (AFCs) were also enriched (second column). These results indicated that artTLOs possess a remarkable property to efficiently accumulate memory T and memory B cells as well as antigen-specific AFCs that had been recruited from the recipient Balb/c mouse lymphoid tissues during lymphocyte cell trafficking between recipient mouse and artTLO tissue through blood vessel. The artTLOs, which were constructed in antigen-primed recipients by applying the gel-trapped chemokines, work as an efficient reservoir for memory T and B cells and also for antigen-specific AFCs.

### artTLOs Function as Highly Active Immune Tissues Following Transplantation into Immuno-Compromised Recipients

**Figure 3:**
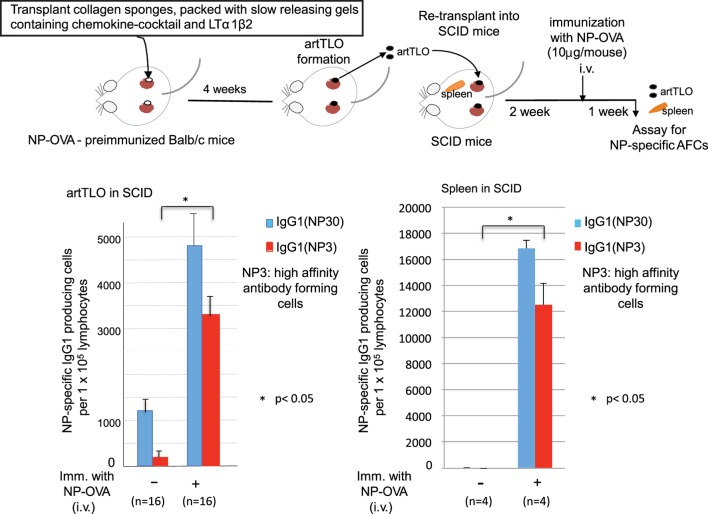
**Enrichment of antigen-specific high-affinity antibody-producing cells in artTLO upon transplantation into immunodeficient SCID mice**. ArtTLO were first formed in eight NP-OVA preimmunized Balb/c mice as described in Figure 2. Four artTLO (two artTLO in each kidney) in one mouse were constructed. Then, artTLO were individually extirpated and collected from Balb/c mice and re-transplanted into renal subcapsular space of eight SCID mice. Four artTLO were re-transplanted into each SCID mouse. Two weeks after re-transplantation, four of artTLO-carrying SCID mice were intravenously immunized with 10 μg NP-OVA. The other four SCID mice were not immunized. One week after, artTLO and recipient spleen were extirpated individually from SCID mice and single cell suspensions were prepared. Numbers of high affinity as well as total IgG class anti-NP antibody-forming cells (AFCs) in individual artTLO and in spleens of recipient SCID mice were counted by an immunospot analyzer. Numbers of AFCs were calculated from 16 individual artTLO for each group.

artTLOs, which had been formed in antigen (NP-OVA)-preimmunized mice and then excised, were re-transplanted into renal subcapsular spaces of immune-deficient SCID mice having no mature T and B lymphocyte (Figure 3). Two weeks after transplantation, half of the SCID mice carrying artTLO were intravenously immunized with the same antigen. The other half of the SCID mice carrying artTLO remained un-immunized. One week later, artTLOs and spleen of recipient SCID mice were removed. Cell suspension was prepared from each individual artTLO and spleen. IgG1 class anti-NP-specific AFCs in artTLOs as well as recipient SCID spleens were counted as shown in Figure 3. Total (NP30) and high affinity (NP3) of NP-specific AFCs were measured. Low but significant numbers of anti-NP AFCs were detected in re-transplanted artTLO in non-immunized SCID mice. They were remnant of the AFCs that had been migrated from the first NP-OVA preimmunized Babl/c mouse. On the other hand, numbers of antigen (NP)-specific high affinity as well as total IgG class antibody-producing cells were remarkably increased in re-transplanted artTLOs upon immunization (Figure 3, left side), indicating that secondary immune response was efficiently induced by memory B and T cells that had been accumulated in the re-transplanted artTLO as shown in Figure 2. Anti-NP AFCs were hardly detected in spleen of artTLO-re-transplanted SCID mice without immunization of SCID mice with NP-OVA (Figure 3, right side), indicating that no migration of AFCs occurred in the artTLO-carrying SCID mice even though the presence of empty space in SCID mouse spleen for lymphoid cells and even though that lymphocyte could freely communicate between artTLO and SCID mouse immune tissues through blood vessels. Surprisingly, extraordinarily large numbers of anti-NP AFCs appeared in spleens of the artTLO-carrying SCID mice as shown in Figure 3 (right side), suggesting that NP-specific memory B cells and T cells are migrating from artTLO into empty SCID spleen and they quickly maturate and explosively expand into NP-specific AFCs in the empty space in SCID mouse spleen upon antigen stimulation. Thus, artTLOs constructed by the present gel-trapped lymphoorganogenic chemokines are effective immune tissues, especially in immune-compromised hosts, in which the artTLO could induce a strong specific immune response upon antigen stimulation.

## Discussion

The major conclusion of the data detailed above is that immunologically highly active and transplantable artTLOs can be generated in the absence of LTo cells in mice by providing suitable factors that are usually secreted from LTo upon stimulation by lymphoid tissue inducer cells (LTi). We successfully constructed immunologically functional artTLO by applying slow-releasing gels containing combination of four chemokines (CXCL12, CXCL13, CCL19, and CCL21) and sRANKL together with lymphotoxin α1β2 protein. We propose that the strategy applied here may lead the way to the generation of artTLOs not only in mouse but also in human to ameliorate untreatable diseases as varied as severe infection (sepsis), primary and secondary immune deficiency syndromes, autoimmunity, and a large number of clinically important chronic inflammatory diseases, including atherosclerosis, rheumatoid arthritis, and inflammatory bowel and brain diseases (37, 39, 40, 54–62). It is well known that T cell immune responses are actively induced in tumors (63). However, tumors have evolved to acquire immunosuppressive mechanisms or they apply other immune evasion mechanisms. Recent advances in checkpoint therapy for cancers will be an effective strategy to overcome some of these hurdles (64, 65). The artTLO may provide the machinery to assist checkpoint therapy for cancers. Also, atrophy of primary and SLOs, which occur during aging associated with immuno-senescence (66, 67), gives rise to fatal infectious diseases in the elderly. The artTLO may play a role in reinforcement of immune function in the elderly. Moreover, artTLOs should be examined for their ability to counteract the compromised immune system in patients who receive radiation therapy or chemotherapy. We further envisage application of artTLO in patients that undergo hematopoitic stem cell transplantation during conditioning regimens, including non-myeloablative conditioning to overcome a window in time of severely compromised immune function, since the immune cells in the artTLO rapidly may expand and maturate in the patients much as they would in a SCID mouse as shown in Figure 3.

Since artTLOs in the present study are formed on the scaffold of a collagen sponge, it is easily removable and transplantable. As the characteristics of biomaterials are important in tissue engineering strategies, a collagen sponge was applied. In order to efficiently generate artTLOs, the scaffold should be carefully prepared to mimic the natural environment of TLO neo-genesis. Scaffolds for the synthesis of immune tissues are required to allow the LTo cells and respective immune cell populations to organize themselves to create microenvironments that allow artTLO neo-genesis. Such scaffolds should also maintain the three-dimensional structure that allows immune cells to move effectively for both optimal recruitment and egression (through the newly formed HEVs, blood vessels, and lymphatics), and maintain a reservoir of soluble factors, such as chemokines and cytokines expressed by stromal cells. A number of synthetic biomaterials have been developed, which are all able to duplicate the three-dimensional microenvironments that are provided by natural extracellular matrices, such as fibril or non-fibril collagen, proteoglycans, matrix cellular proteins (68), and their hydrogels (69). It has been demonstrated that structurally engineered macro-porous scaffolds, which combine polyethylene glycol hydrogels with collagen, support T cell, and DC migration (70). The first *in vivo* synthesis of artificial lymphoid tissues was achieved by using a porous biocompatible collagen matrix, prepared from the bovine Achilles tendon (referred to as a collagen sponge) (49). It has a non-homogeneous pore size ranging from 50 to 300 μm. A key in lymphoid tissue engineering is to properly modulate and mimic dynamics of lymphocyte trafficking. In addition, it is important to recruit the appropriate immune cells to the lymphoid tissues. Finally, the gradients of the soluble factors secreted from stromal cells need to be adjusted. Biomaterials that release soluble factors simultaneously not only uniformly but also gradually with temporal differences have been described (71). In the present study, Medgels, which consist of collagen gels, were applied. Medgels maintain and slowly release soluble protein molecules. The 3D scaffold in which cell-specific chemokines/cytokines are geometrically positioned and fixed based on the histological patterns are expected to be advantageous for the formation of more sophisticated and functional artTLOs. In order to do so, a manufacturing approach using 3D bioprinters should be attempted.

There has been much progress in the establishment and analysis of humanized mice (72–77). As a result, humanized mice will be utilized as human disease models. They can be experimentally manipulated and used to directly study infectious diseases, immunological disorders, and cancers (78–80). These model systems could be suitable candidates for generating human artTLOs in future by applying the present slow-releasing gels containing chemokine cocktail and lymphotoxin-α1β2.

## Conclusion

Tertiary lymphoid organs are unique lymphoid tissues in which interaction of antigen-presenting cells with effector T- and B-lymphocytes is organized and followed by induction of protective adaptive as well as innate immune responses. The synthesis of artificially constructed TLO tissues (artTLOs) that function as the effective substitutes for SLOs may be a promising novel strategy to treat both local and systemic infections, autoimmune diseases, and cancer. Development of functionally active human artTLOs or similar devices is expected in the near future. In this report, we have attempted to construct artificially made functional TLOs by applying gel-trapped lymphorganogenic soluble factors, instead of using lymphoid tissue stromal cells. They showed a remarkable immune function especially as a reservoir of antigen-specific memory B and T cells.

## Materials and Methods

### Antibodies and Reagents

Fluorescein-, phycoerythrin-, or biotin- labeled anti-B220 (clone:RA3-6B2), anti-CD3 (Clone:145-2c11), anti-Thy1.2 (clone:30-H12), anti-CD11c (clone:N418), anti-CD21/35 (clone:7G6), anti-FDC-M1 (clone:FDC-M2) were obtained from BD Pharmingen. Anti-ER-TR7 was from Abcam plc., anti-PCAM-1 (clone:390), and anti-PNAd (clone:MECA-79) were from Biolegend. Anti-IgG1 and goat anti-hamster IgG and fluorescein- or phycoerythrin-labeled streptavidin were all purchased from BD Bioscience. NP-OVA and NIP-BSA were purchased from Bioresearch Technology. Lymphotoxin α1β2 was purchased from R&D System. CXCL13 (cat no.:300-47), CCL19 (cat. No:300-29B), CCL21 (cat. No:300-35), CXCL12 (cat no:300-28A), and sRANKL (cat. No:310-01) were purchased from Peprotech. Medgel was obtained from MeDGEL Co., LTD (Japan).

### Mice

Balb/cAnNcrj mice and SCID mice (C.B.-17/IcrCrj-scid/scid) were purchased from Japan SLC, Inc. All mice were housed under specific pathogen-free condition in the animal facility of Medical Research Institute, Kitano Hospital. All experiments described herein were approved by the Kitano Hospital animal use committee and were performed in accordance with the applicable guidelines and regulation.

### Immunization

For pre-immunization, 100 μg NP15-OVA precipitated in alum was injected i.p. into 7- to 10-week-old Balb/c mice. Four or more weeks after immunization, mice were used as donors for the generation of artTLO.

### Synthesis of artTLOs

Since Medgel releases the protein (chemokines, lymphotoxin) very slowly *in vivo* and it is difficult to determine the optimal dose of each protein for *in vivo* formation of lymphoid tissues, large excess amounts of chemokine or lymphotoxin were added to Medgel. Twenty-microliter solution of each kind of soluble factor (CXCL12, CXCL13, CCL19, CCL21, LTα1β2, and sRANKL; 100 μg/ml each) was added to dry powder of Medgel (400 μg) in 1.5 ml microtubes followed by an incubation for 2 h at 37°C. Then, all soluble factor-containing Medgels were once mixed in a microtube. About 50 μg of the mixtures of Medgels was absorbed into a collagen sponge piece (2 × mm × 2 × mm square, CS-35; KOKEN). Approximately 30–40 Medgel-adsorbed collagen sponges were prepared at once and transplanted into renal subcapsular space of 8–10 adult (7- to 10-week-old) Balb/c mice (4 pieces in one mouse). The artTLOs were constantly formed 3 weeks after transplantation.

### Immunohistochemical Staining

artTLOs and lymphoid tissues from recipient mice were embedded in Tissue-Tek OCT compound (SAKURA FINETEK), and snap frozen in liquid nitrogen. Five-micrometer-thick cryostat sections were prepared and placed on APS-coated glass slides (Matsunami Glass Ind. Ltd.). Sections were fixed with cold acetone for 5 min, dried, and kept at −80°C until use. After blocking with 5% normal rat serum and 1% BSA in TBS-T (Tris-buffered saline with 0.005% Tween20) for 1 h at 20°C, sections were incubated for 1 h at 20°C with appropriate antibodies or streptavidin-fluorochrome reagents diluted in blocking buffer and washed with PBS three times every 5 min.

### ELISPOT for Measurement of NP-Specific IgG1 and IgM Antibody-Forming Cells

The frequency of high- and low-affinity NP-specific AFCs among cells collected from artTLOs or spleen cells from donor mice was measured by ELISPOT using NP3-BSA- and NP30-BSA-coated filter paper for low-affinity AFC, respectively, as shown previously (50). Hydrophobic PVDF filters on MultiScreenIP Filter Plates (MAIPS4510, Millipore) were coated with 50 μg/ml NP3-BSA, NP30-BSA, or BSA in PBS at 4°C overnight, and then blocked with 1% BSA in PBS. Cells (0.2–1 × 10^5^ cells/well) were incubated for 2 h at 37°C and washed once with PBS containing 50 mM EDTA, twice with TBS-T, and once with PBS. After washing, filters were visualized with BCIP/NBT (Chemical International) and AEC (BD Biosciences-Pharmingen). Numbers of AFCs were counted by ImmunoSpot Analyzer (C.T.L.).

### Statistics

All the statistical analysis were performed by using an unpaired two-tailed Student’s test. A *P*-value of less than 0.05 was considered significant.

## Studies Involving Animal Research

Animal research in this review were approved by the ethics committee of The Tazuke-Kofukai Medical Research Institute and Kitano Hospital, Osaka, Japan, and carried out in accordance with the recommendation of animal research guidelines issued from the ethics committee.

## Author Contributions

YK performed all experiments. TW planned experiments and wrote the manuscript.

## Conflict of Interest Statement

The authors declare that the research was conducted in the absence of any commercial or financial relationships that could be construed as a potential conflict of interest.
